# Verbalization of emotional states by children with special educational needs

**DOI:** 10.1192/j.eurpsy.2023.421

**Published:** 2023-07-19

**Authors:** A. Akhmetzyanova, T. Artemyeva, T. Korniychenko, I. Dzhurabaeva, Z. Egorova

**Affiliations:** Kazan Federal University, Kazan, Russian Federation

## Abstract

**Introduction:**

The degree of success and effectiveness of the child’s socialization largely depends on the timely formation of social emotions, the ability to understand the emotional states of the participants in the interaction and manage their emotions.

**Objectives:**

studying the features of understanding the emotional states of peers and adults by children of preschool age with special educational needs.

**Methods:**

The study involved 227 children aged 5-7 attending educational institutions: 95 children without developmental disorders; 73 children with severe speech disorders; 9 children with motor disorders; 25 children with visual impairment (strabismus, amblyopia, astigmatism); 15 children with hearing impairment (3rd and 4th degree sensorineural hearing loss); 10 children with autism spectrum disorder. The “Emotional Faces” method (Semago) and the method of studying the child’s understanding of tasks in situations of interaction (Veraksa) were used.

**Results:**

Tasks for the categorization of emotional states cause difficulties in children with speech disorders, since they require a certain mastery of vocabulary for the designation of emotional states. As a result of limited communication in children, there is a lack of understanding of the meaning, causes and motives of the actions of other people, as well as the consequences of their actions, their impact on others.

Preschool children with motor disabilities are inferior to peers without developmental disabilities in accurate verbalization of emotional states, manifested in a primitive description of emotions.

Visually impaired preschool children do not have sufficiently clear ideas about socially acceptable actions in communication situations, about ways of expressing relationships with peers and adults.

Children with hearing impairment better understand the emotional states of their peers than the states of adults, but they do not know how to show their attitude towards their peers. Difficulties in verbalizing emotions are observed.

Children with autism spectrum disorder experience significant difficulties in recognizing various situations of interaction, isolating tasks and requirements set by adults in these situations; children practically did not try to depict an emotion, having difficulty in differentiating it.

**Conclusions:**

The research confirmed the assumption that children with disabilities have significant difficulties in differentiating similar emotions, they do not accurately determine the emotional state of their peers and people around them. This paper has been supported by the Kazan Federal University Strategic Academic Leadership Program.

**Disclosure of Interest:**

None Declared

